# Ultrafast Carrier and Lattice Dynamics in Plasmonic Nanocrystalline Copper Sulfide Films

**DOI:** 10.1002/lpor.202000346

**Published:** 2021-01-21

**Authors:** Anton Yu. Bykov, Amaresh Shukla, Mark van Schilfgaarde, Mark A. Green, Anatoly V. Zayats

**Affiliations:** ^1^ Department of Physics and London Centre for Nanotechnology King's College London London WS2R 2LS UK; ^2^ Prof. M. van Schilfgaarde National Renewable Energy Laboratory Golden Colorado 80401 USA

**Keywords:** coherent optical phonons, copper sulfide, nonequilibirium processes, plasmonics, ultrafast hot‐carrier dynamics

## Abstract

Excited carrier dynamics in plasmonic nanostructures determines many important optical properties such as nonlinear optical response and photocatalytic activity. Here it is shown that mesoscopic plasmonic covellite nanocrystals with low free‐carrier concentration exhibit a much faster carrier relaxation than in traditional plasmonic materials. A nonequilibrium hot‐carrier population thermalizes within first 20 fs after photoexcitation. A decreased thermalization time in nanocrystals compared to a bulk covellite is consistent with the reduced Coulomb screening in ultrathin films. The subsequent relaxation of thermalized, equilibrium electron gas is faster than in traditional plasmonic metals due to the lower carrier concentration and agrees well with that in a bulk covellite showing no evidence of quantum confinement or hot‐hole trapping at the surface states. The excitation of coherent optical phonon modes in a covellite is also demonstrated, revealing coherent lattice dynamics in plasmonic materials, which until now was mainly limited to dielectrics, semiconductors, and semimetals. These findings show advantages of this new mesoscopic plasmonic material for active control of optical processes.

## Graphical abstract

Excited carrier dynamics in plasmonic nanostructures determines many important optical properties. Here a comprehensive study of equilibrium and non‐equilibrium hot carrier dynamics in mesoscopic plasmonic covellite nanocrystals is reported, which exhibit a much faster carrier relaxation than traditional plasmonic materials. Furthermore, coherent lattice dynamics in plasmonic materials is demonstrated and analyzed.

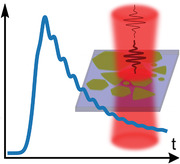

## 1 Introduction

Utrafast dynamics of photoexcited electrons in plasmonic metals is a key process underpinning various practical applications ranging from ultrafast switching^[^ ^1^, ^2^, ^3^ ^]^ to hot‐electron‐induced photochemistry and sub‐band photodetection.^[^ ^4^, ^5^, ^6^, ^7^, ^8^, ^9^ ^]^ The hot‐electron dynamics even in prototypical plasmonic metals, such as Au and Ag is determined by a plethora of nonequilibrium processes involving complex interactions between electronic and lattice subsystems,^[^ ^10^, ^11^ ^]^ as well as various nonlocal diffusive and/or ballistic transport phenomena.^[^ ^12^, ^13^, ^14^, ^15^ ^]^ On an ultrashort timescale, a nonequilibrium electron distribution has to be considered even in the most simplified experimental configurations.^[^ ^16^, ^17^, ^18^, ^19^, ^20^, ^21^ ^]^ Recent advances in plasmonic photochemistry^[^ ^4^, ^5^, ^9^ ^]^ facilitated development of complex models for hot‐carrier excitation and decay.^[^ ^19^, ^22^, ^23^ ^]^ Transient optical processes are important for studies of photocatalysis^[^ ^24^ ^]^ since the lifetime of the photoexcited hot‐carrier population can be linked to photocatalytic performance.^[^ ^25^ ^]^

Control over the hot‐carrier dynamics can be achieved either by geometrical means (nanostructuring) or by choosing materials with different electron–electron and electron–phonon scattering rates. In the former case, the size of nanostructures and their anisotropic shape influences the nonequilibrium and equilibrium dynamics.^[^ ^26^, ^27^, ^28^, ^29^ ^]^ In the latter case, the free‐electron concentration is the important parameter as it affects the electron scattering.

While traditional metals have very high electron concentrations of the order of 6 × 10^22^ cm^−3^, in some other plasmonic materials such as nitrides,^[^ ^30^ ^]^ heavily doped semiconductors, transparent conductive oxides and sulfides,^[^ ^31^ ^]^ the free carrier concentration can be adjusted during growth or by applying an external electric bias.^[^ ^32^, ^33^ ^]^

Here we study peculiarities of the transient dynamics in a new mesoscopic p‐type plasmonic material consisting of covellite (copper monosulfide, CuS) nanocrystals with more than order of magnitude lower free‐carrier concentration (≈3 × 10^21^ cm^−3^) than in Au or Ag. Copper monosulfide is a naturally occurring layered metal chalcogenide mineral. At room temperature, it exhibits a hexagonal (space group P6_3_/mmc) structure consisting of alternating CuS_3_ and CuS_4_ units, while below 55 K, it undergoes second‐order structural phase transition into the orthorombic phase^[^ ^34^ ^]^ which is a general trend shared among all copper sulfides and CuS${}_{1-x}$Se_*x*_ solid solutions.^[^ ^35^ ^]^ Due to the partially filled valence band, covellite has metallic conductivity due to the holes associated mainly with the 3p orbitals of sulfur and to lesser extent Cu 3d and S 3s orbitals.^[^ ^36^, ^37^ ^]^ Because of its crystal structure, covellite has an anisotropic conductivity at low frequencies^[^ ^38^, ^39^ ^]^ that eventually evolves into two‐dimensional conductivity at low temperatures.^[^ ^40^ ^]^ At optical frequencies, the anisotropy makes it a natural hyperbolic optical material^[^ ^41^ ^]^ with the epsilon‐near‐zero behavior in the near‐infrared wavelength range.

Covellite can be chemically synthesized in a form of mesoscopic nanoplatelets which exhibit localized surface plasmons in the near‐IR (in the spectral range 1.2–1.6 µm),^[^ ^42^ ^]^ which makes it a plasmonic material complementary to noble metals. Use of CuS as a high‐performance catalyst for oxygen‐reduction reactions has also been demonstrated.^[^ ^43^ ^]^ Both the low free‐carrier concentration and mesoscopic nature of the nanocrystals (typical thickness approximately 4 nm) may affect the transient carrier dynamics in the optical response of a covellite.

In this article, we present a comparative optical pump‐probe investigation of both monolayer nanocrystalline films and a bulk covellite on the sub‐picosecond time scales, highlighting both nonequilibrium and equilibrium dynamics and a presence of coherent phonon modes. Much faster carrier relaxation dynamics is observed than in traditional plasmonic materials explained by a low free‐carrier concentration, which is further accelerated in mesoscopic films due to the reduced Coulomb screening. The unusual for plasmonic materials coherent lattice dynamics is also studied.

## 2 Results and Discussion

### 2.1 Hot‐Hole Dynamics

After a short optical pulse excites electrons in metals, a non‐equilibrium step‐like hot‐electron distribution is formed in the energy range ±ℏω around the Fermi level. Within the standard framework for hot‐electron relaxation in metals,^[^ ^16^ ^]^ this non‐equilibrium population decays within the first few hundred femtoseconds due to a sequence of elastic electron–electron collisions resulting in the equilibrium population of electrons with the temperature higher than the lattice temperature. These thermalized hot electrons cool down with emission of phonons. The latter process has been successfully treated assuming the heat exchange between two quasi‐equilibrium thermodynamic ensembles: hot electrons and lattice in what is now known as two‐temperature model (TTM).^[^ ^10^, ^11^ ^]^ In the next sections, we first discuss the relaxation dynamics of the thermalized carriers and then their initial nonequilibrium dynamics.

#### 2.1.1 Hot‐Carrier Cooling

**Figure 1:**
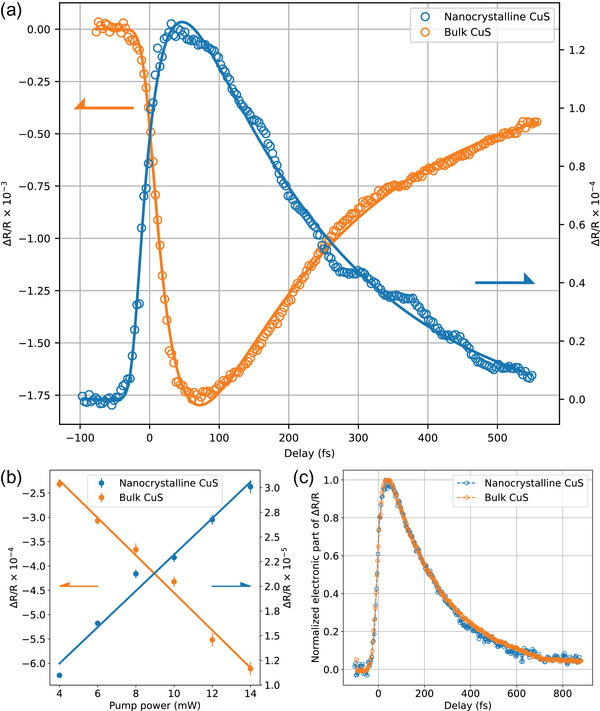
a) Transient reflection dynamics, b) pump‐power dependences of the amplitude of the induced reflection, and c) comparison of the hot‐hole relaxation for the bulk covellite and the mesoscopic CuS film.

For copper monosulfide, after photoexcitation we expect fast relaxation of optical constants governed by equilibrium and non equilibrium dynamics of hot holes. **Figure** **1**a shows typical pump‐probe traces measured for bulk and mesoscopic CuS. Both transient responses exhibit similar trend with relatively sharp rise time and slow sub‐picosecond‐scale decay. Superimposed with the decay curve, the oscillatory behavior is clearly visible in mesoscopic films. These oscillations correspond to coherently excited optical phonon modes which will be discussed in detail later. For moderate pump powers, the amplitudes of the transient reflectivity change for both samples depends linearly on the pump power (Figure 1b). The opposite signs of the transient response can be understood in terms of conventional thin film optics. For the bulk crystal the transient reflectivity is dominated by the transient modification of the imaginary part of a CuS dielectric function. On the other hand, for thin films, with the thickness comparable to optical penetration depth, multiple reflections have to be considered in a standard way so that the resulting reflection coefficient is ${|{\left(1+r_{12}r_{23}e^{2ikd}\right)}^{-1}|}^{2}$, where r_12_ and r_23_ represent the amplitude reflection coefficients on air/CuS and CuS/substrate interfaces, respectively. In the latter case, the transient modification of a real part of CuS dielectric constant becomes dominant. Realistic calculations require the precise knowledge of optical constants of an “effective medium” formed by nanoparticles in the mesoscopic CuS film and are not presented here. While optical constants even of bulk CuS are not well known and their determination from spectroscopic ellipsometry is complicated due to anisotropic nature of CuS, for qualitative estimates, we used the ab initio derived dielectric function^[^ ^41^ ^]^ and its fit with the Drude–Lorenz model (Figure 4b). It is clear that, in the spectral range of the pump‐probe pulses, the Drude contribution is dominant. Using the fitted dielectric function, it can be qualitatively shown that bulk CuS and a uniform 4 nm thick film of CuS on glass produce transient responses of opposite signs when the increase of the Drude damping parameter emulates the excitation.^[^ ^44^ ^]^

**Figure 2:**
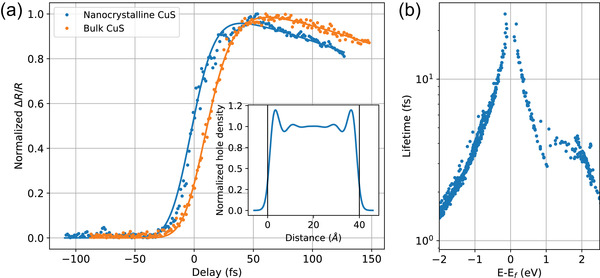
a) Comparison of the transient reflection risetime in the bulk covellite and the mesoscopic CuS film. Inset shows the free‐carrier density profile in a thin (4 nm) CuS slab normalized to the bulk carrier density. b) Hot‐carrier lifetimes as a function of energy for CuS calculated with the GPAW code (See Experimental Section).

**Figure 3:**
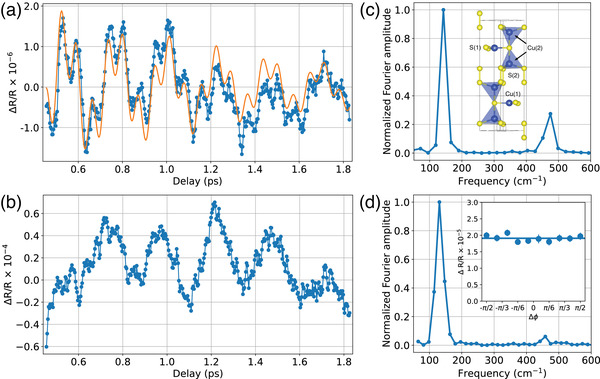
The transient reflection dynamics due to the excitation of coherent optical phonons in a) the mesoscopic CuS film and b) the bulk covellite: (blue) experimental data, (orange) biharmonic fit using the phonon frequencies in (c). c) The spectrum of optical phonons obtained by the fast Fourier transform of the time dependence in (a), revealing two modes. Inset shows the crystal structure of covellite. d) The spectrum of optical phonons obtained by the fast Fourier transform of the time dependence in (b), revealing mainly the low frequency phonon mode. A much weaker peak is visible around 475 cm^−1^. Inset shows the dependence of the amplitude of the lowest energy phonon mode (measured in a bulk covellite) on the angle between polarizations of the pump and probe beams.

**Figure 4:**
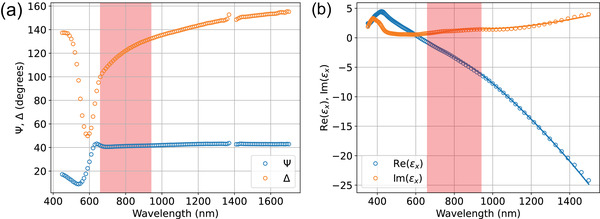
a) Spectra of Ψ and Δ obtained from the spectroscopic ellipsometric measurements of the CuS nanocrystalline film. b) In‐plane dielectric constant of CuS: (circles) obtained from the simulations in ref. [41] and (lines) the fit with the Drude–Lorenz model. Highlighted region corresponds to the spectral width of the broadband fs pulses used in the pump‐probe experiments. Please note that the connection between the dielectric constant of the bulk CuS and the nanocrystalline film require the development of the effective medium model which is beyond the scope of this work.

The temporal shapes of the pump‐probe traces do not change much with a pump power (see Supporting Information), and the decay time shows only weak dependence on the pump fluence which is consistent with the relaxation of hot carriers in metals rather than dynamics of photogenerated carriers in semiconductors and semimetals. Even though nominally the pulses used contain photons with the energies above the optical band gap of CuS, as confirmed by measurements with a tuneable probe (see Supporting Information), we do not expect the photogenerated carriers to contribute to the dynamics due to both a weak excitation regime realized in the experiment (fluence below 0.2 mJ cm^−^^2^) and high (compared to semiconductors and semimetals) intrinsic free‐carrier concentrations. The decay time determined here in the limit of low fluences is of the order of 200 fs.

Even though the amplitudes of the traces are different in both sign and magnitude, it is still possible to quantitatively compare the hot‐hole decay time in nanocrystalline films and bulk covellite crystals. Figure 1c shows normalized monotonic decay traces with oscillatory and lattice contribution subtracted. It can be seen that the hot‐carrier relaxation processes are very similar in the bulk and mesoscopic CuS. This is in a sharp contrast to the previous experiments with noble metal films where an order of magnitude slower relaxation of the induced optical changes were observed for thin films.^[^ ^45^ ^]^ Indeed, in good metals, heat dissipation into the bulk competes with the electron–phonon interaction as it happens on similar few‐picosecond timescales, resulting in a gradually faster relaxation for films thicker than the optical penetration depth.

Due to a lower free carrier concentration in CuS (3 × 10^21^ cm^−3^ compared to 5.9 × 10^22^ cm^−3^ in gold^[^ ^46^ ^]^), the diffusion contribution is expected to be suppressed. Not only does this make CuS a cleaner experimental system for studying hot‐carrier dynamics but also opens the way to investigate other delicate effects in nanocrystals. One of the important questions that arise when comparing the properties of bulk and mesoscopic systems and nanocrystals in solutions is the role of the surfaces and interfaces. This may include, but is not limited to, surface trap states,^[^ ^47^ ^]^ surface relaxation and reconstruction, role of adsorbates and an environment.^[^ ^48^ ^]^ In this context, we conclude that the striking similarity between the transient response of two different systems with much different surface to bulk ratio rules out any surface effects as well as quantum confinement as a source of observed hot‐carrier population decay.

The observed decay with approximately 200 fs time constant is, therefore, associated with local relaxation (not related to diffusion or ballistic transport) in the photoexcited hot‐hole gas based on agreement with the bulk covellite. This indicates that at these time scales and excitation conditions, bulk properties of a covellite can be used to describe the optical response of CuS nanoparticles, which is consistent with the localized surface plasmon measurements.^[^ ^41^ ^]^

#### 2.1.2 Nonequilibrium Dynamics

Another important characteristic of hot‐carrier dynamics is the initial thermalization within the nonequilibrium hot‐carrier ensemble. Thermalization time describes the lifetime of hot holes at states far from the Fermi level, which in turn determines the probability to tunnel into the states of the adjacent adsorbates before their energy is dissipated and is of paramount importance for surface chemistry.

Theoretical framework for ultrafast thermalization in a photoexcited Fermi gas is well established with different degrees of complexity, from the simple rate equation formalism for a nonequilibrium part of the carrier distribution in terms of occupation numbers^[^ ^16^ ^]^ to numerical solutions of the Boltzmann kinetic equation.^[^ ^16^, ^19^ ^]^ In our case, due to the broadband nature of the measured response, in order to determine the electron–electron scattering rate responsible for the thermalization process the initial risetime of the transient reflectivity changes ΔR/R was fit to a phenomenological expression^[^ ^16^, ^27^ ^]^:

(1) $$\frac{\Delta R}{R}\propto \Theta \left(t\right)\left(1-e^{-t/\tau_{\text{th}}}\right)e^{-t/\tau_{e-\text{ph}}}⊗G\left(t\right)$$

where Θ(t) is the Heaviside step function, G(t) is the pump‐probe pulse cross‐correlation function and $\tau_{\text{th}}$ and $\tau_{e-\text{ph}}$ are the time constants determining the rates of thermalization in the hot‐hole ensemble and cooling due to electron–phonon interaction. Equation (1) together with the additional rising exponent for the lattice contribution (with time constant equal to $\tau_{e-\text{ph}}$) have been used to generate exponential fits in Figures 1a and **2**. Recovered thermalization time for bulk CuS is estimated to be approximately 18 fs which is about an order of magnitude shorter than the typical values for the bulk noble metals.

As a simple estimate, a homogeneous free‐electron gas model provides understanding of this difference. Since electron–electron scattering events are governed by the (screened) Coulomb interaction, the thermalization time is expected to rise with the bulk carrier density and core electron screening^[^ ^27^, ^28^ ^]^ as

(2) $$\tau_{\text{th}}\propto n_{e}^{5/6}\varepsilon_{c}^{1/2}$$

where $n_{e}$ is the electron concentration and $\varepsilon_{c}$ is the core electron contribution to the bulk static dielectric constant. Since the dependence on a free carrier concentration alone gives a factor around 12, we do expect much faster thermalization dynamics in CuS than in noble metals.

Quantitatively, electron–electron scattering times can be evaluated from the imaginary part of a quasiparticle self‐energy, calculated, for example, in the GW approximation. We perform the calculations for CuS in the local density approximation and a single‐shot G0W0 approach. While not properly self‐consistent, this approach has been successfully used to evaluate the electron–electron scattering in metals.^[^ ^49^ ^]^ It should be noted that, while nominally the electron–electron scattering depends on the energy of hot electrons as ${\left(E_{i}-E_{F}\right)}^{2}$, it is mainly the longer processes which happen in the vicinity of the Fermi level that determine the experimentally observable decay time. Figure 2b shows that closer to the Fermi level, the hot‐electron scattering times are around an order of magnitude smaller than those for noble metals,^[^ ^49^ ^]^ which is comparable to the difference between thermalization times obtained from the pump‐probe experiments in CuS (this work) and in the measurements for noble metals.^[^ ^27^, ^28^ ^]^ We, therefore, conclude that much faster thermalization in a covellite compared to conventional metals is due to the reduced Coulomb screening in a much less dense free‐carrier gas.

For mesoscopic CuS films of 4 nm thickness, the thermalized state is reached at even shorter timescales of about 14 fs. This can be explained with the reduced screening near the edges of the particle due to “electron gas spillout”,^[^ ^50^ ^]^ similar to silver and gold nanoparticles.^[^ ^27^, ^28^ ^]^ A dynamic calculation of hot‐carrier scattering in this geometry is computationally demanding; however, these effects can be taken into account in the static screening approximation by weighting the dependence in Equation (2) with the locally varying background carrier density^[^ ^28^ ^]^:

(3) $$\frac{1}{\tau_{\text{th}}}\propto \frac{\int_{-\infty }^{\infty }\tau_{\text{th}}^{-1}\left[n_{e}\left(z\right)\varepsilon_{c}\left(z\right)\right]n_{e}\left(z\right)dz}{\int_{-\infty }^{\infty }n_{e}\left(z\right)dz}$$

The integrals in Equation (3) for a 4 nm thick CuS slab were evaluated with the electron distribution profile derived from the simplistic jellium model^[^ ^51^ ^]^ and the static dielectric functions taken as 3.7 for a SiO_2_ substrate, a bulk CuS permittivity inside the mesoscopic slab, and 1 for an air superstrate (Figure 2, inset). The resulting relation between the bulk and mesoscopic thermalization times $\tau_{NC}\approx 0.7\tau_{\text{bulk}}$ is consistent with the measured experimental values.

### 2.2 Lattice Dynamics

Absorption of ultrashort optical pulses by solid media may lead to the generation of coherent optical phonons. These excitations have been extensively studied in various materials, most notably semiconductors and semimetals^[^ ^52^, ^53^, ^54^, ^55^, ^56^ ^]^ but observations of coherent phonons in metals are scarce^[^ ^57^ ^]^ and only limited to Zn and heavy rare‐earth elements such as Gd and Tb.^[^ ^58^, ^59^, ^60^ ^]^ The observation of oscillatory components in the transient reflection (Figure 1) is, therefore, promising from both the fundamental perspective of coherent phonon dynamics in plasmonic systems and for the development of a new experimental tools for studies of interaction between hot carriers and phonons.

The unit cell of a covellite crystal contains 14 atoms and, therefore, a total of 39 optical phonon modes are supported in the crystal. Since usually only Raman active optical phonons are observed in optical pump‐probe experiments, it is instructive to consider their classification. According to the group‐theoretical analysis, the Raman active phonon modes in the centre of the Brillouin zone can be classified as^[^ ^35^ ^]^ $2A_{1g}+2E_{1g}+4E_{2g}$, where A_1*g*_ are the fully‐symmetric optical phonon modes which correspond to the motion of atoms along the c‐axis in the unit cell and E${}_{1g/2g}$ are the modes of lower symmetry. The corresponding Raman tensors are presented in Supporting Information.

It is well established that the most prominent peak in the Raman scattering from a covellite around 475 cm^−1^ corresponds to one of the A_1*g*_ modes formed by motion of S(2) atoms in the 4e sites in the unit cell.^[^ ^35^, ^61^ ^]^ The second fully symmetric mode is not clearly identified in the literature but, based on the symmetry considerations, we believe it corresponds to the vertical motion of Cu(2) atoms in the 4f sites. The remaining modes are “transversal” with respect to the c‐axis and the sample surface normal and are often not observed in the pump‐probe experiments without anisotropic detection.^[^ ^62^ ^]^

**Figure** **3**a,b illustrates the extracted oscillatory components of the transient reflectivity in both the mesoscopic film and the bulk covellite. The spectrum of the observed phonon modes Figure 3c shows that the film supports two coherently excited lattice vibrations with the high frequency one consistent with the S(2)‐S(2) breathing A_1*g*_ mode. It is, therefore, natural to assume that lower frequency mode at around 150 cm^−1^ corresponds to the second A_1*g*_ mode related to the motion of Cu(2) atoms. This low frequency mode is also observed in the bulk covellite crystals and can be correlated with 142 cm^−1^ peak observed in Raman scattering.^[^ ^35^ ^]^ Following the symmetry analysis (see Supporting Information), it is clear that the “transversal“ E_1*g*_ modes cannot be excited with the s‐polarized pump; however, the 4 double‐degenerate E_2*g*_ (8 modes in total) can.

The amplitude of these modes should, however, follow a cos2ϕ dependence on the relative angle between polarizations of the pump and the probe beams. This check was performed by rotating the polarization of the probe beam under the nearly normal incidence (so that all the fields remain in the plane of the sample) on the bulk CuS sample (Figure 3d, inset). The absence of an angular dependence clearly indicates that the low frequency phonon mode is fully‐symmetric and no modes of lower symmetry are excited. These observations, in analogy to the time‐resolved studies of semimetals, make it most probable that the coherent phonons in CuS are excited through displacive mechanism (or resonant impulsive stimulated Raman scattering, as it is referred to in some papers^[^ ^62^, ^63^ ^]^) caused by interaction of the crystal lattice with the photoexcited hot‐hole gas. Another indirect indication of displacive excitation is that the relative amplitudes of the two observed modes do not match the corresponding amplitudes observed in the Raman scattering experiments.^[^ ^35^ ^]^ It is quite remarkable that while the lower frequency mode can be seen in both a bulk covellite and nanocrystals, the higher frequency S(2)‐S(2) phonons are not clearly visible in the bulk covellite. The Fourier transform spectrum shows a weak peak at the expected frequency with the amplitude about an order of magnitude weaker than in a mesoscopic CuS film (Figure 3d). The exact reason for this behavior is beyond the scope of this study. We, however, can theorize that the S(2)‐S(2) motion can be much more sensitive to the surface termination of the crystal than the motion of Cu(2) ions which are ”protected${}^{\prime \prime }$ by the S(1) tetrahedrons and, therefore, the former can be enhanced in thin nanocrystals with large surface to bulk ratio.

## 3 Conclusion

We have shown that ultrafast transient optical response of p‐type plasmonic, bulk and mesoscopic copper monosulfide shows much faster carrier relaxation dynamics than in traditional plasmonic materials, governed by low free‐carrier concentration.

Both nonequilibrium hot‐carrier and lattice dynamics are modified in CuS nanocrystals with reduced dimensions, showing faster thermalization rate due to the reduced Coulomb screening and twice the number of fully symmetric coherent optical phonon modes, compared to the bulk crystal. The electron–phonon scattering dynamics, on the other hand, remains generally unchanged in thin films compared to bulk crystals. The understanding and control of carrier dynamics is important for the development of the applications of CuS nanocrystalls and films in nonlinear optics and photocatalysis as well as basic understanding of hot‐hole dynamics in this unusual p‐type plasmonic material.

## 4 Experimental Section

### Samples

Two types of samples were used for the pump‐probe experiments: colloidal CuS nanocrystals and bulk covellite single crystals. Bulk covellite crystals were grown by 2D Semiconductors and were indigo‐blue monocrystals with 50–200 µm sized flat facets. The bulk CuS crystals and the CuS nanofilms were characterized with spectroscopic ellipsometry and show no optical resonances in the spectral range of the brodband fs pulses used in this study (Figure 4).

**Figure 5:**
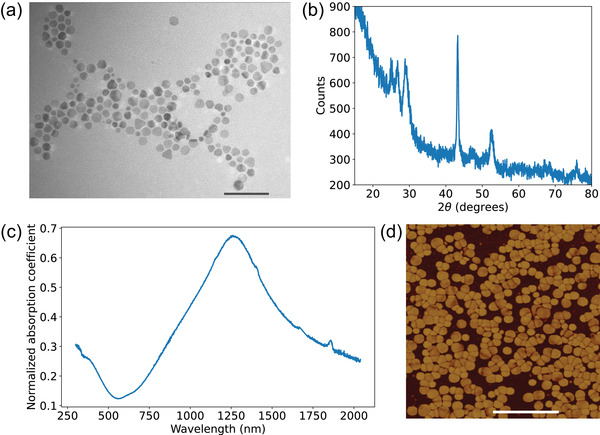
a) TEM image of the as‐prepared CuS nanocrystals; scale bar is 100 nm. b) X‐ray diffraction pattern of the CuS nanoparticles. c) IR absorption spectrum of CuS nanocrystals in chloroform, demonstrating a localized surface plasmon resonance at a wavelength of approximately 1.3 µm. d) AFM phase image of a densely‐packed monolayer of CuS nanoparticles; scale bar is 500 nm.

CuS nanocrystal colloids were prepared using the wet synthesis following the procedure described in ref. [64]. As a starting point, 0.5 mmol of CuCl was mixed with 6 mmol of oleylamine, 12 mL of octadence and 2 mL of oleic acid in a three neck flask. The solution was degassed multiple times and then continuously stirred under vaccum for 10 min. The solution was then gradually heated to 190° C, under vigorous stirring, under an inert atmosphere when it turned pale yellow. The temperature was then reduced to 180° C. An already prepared solution of 64 mg sulphur in 2 mL octadecene degassed and heated to 50° C was then injected immediately into the Cu precursor solution. The color changed to dark green immediately and the temperature was observed to drop to 160° C which was again stabilized to 180° C. The post‐injection reaction was allowed to proceed for 10 min and then the solution was allowed to cool to room temperature. The solution was then mixed with an equal volume of ethanol with acetone added dropwise until flocculation was observed. The mixture was then centrifuged at 4500 rpm for 5 min. The obtained precipitate was washed with methanol multiple times and then dissolved in chloroform. Multiple cycles of the above cleaning procedure were performed to clean the nanoparticles from extra surfactants and residual sulphur. Finally, the precipitated CuS nanoparticles were dissolved in chloroform. As‐synthesized CuS nanoparticles exhibit the X‐ray diffraction (XRD) peaks comparable with those attributed to a bulk covellite with additional broadening due to the confined geometry (**Figure** **5**b). Energy‐dispersive X‐ray spectroscopy (EDX) confirms the chemical composition of copper monosulfide. The colloids were green in color and IR transmission measurements revealed a strong plasmonic absorption peak centered around 1.3 µm (Figure 5c). TEM imaging showed that the prepared nanoparticles were disk‐like hexagons (Figure 5a) similar to reported previously.^[^ ^42^, ^43^, ^64^ ^]^

CuS nanoparticles were deposited on the plain glass substrates using the procedure similar to the one described in ref. [65]. The substrate was cleaned by alternately rinsing in a DI water and immersing in fresh pirahnha solution and heating up to 100° C for 40 min. The substrates were then dried in a nitrogen flow and chemically functionalized through immersion in a (0.04% volume) (3‐mercaptopropyl)trimethoxysilane (MPTMS) solution in toluene and shaken overnight. The functionalization with MPTMS ensured bonding between the nanoparticles and glass substrates as well as allowed for precise control of the thickness of the film. Functionalized substrates were then immersed in a CuS colloid solution and shaken overnight. The samples were subsequently washed in ethanol, ultra‐sonicated in acetone to remove the residual unattached CuS nanoparticles from the surface and dried in a nitrogen flow. For the optical measurements, densely packed monolayer of CuS nanocrystals was used that appears completely transparent when observed with a naked eye. The AFM phase image of the typical CuS monolayer is displayed in Figure 5d.

### Ultrafast Pump‐Probe Measurements

**Figure 6:**
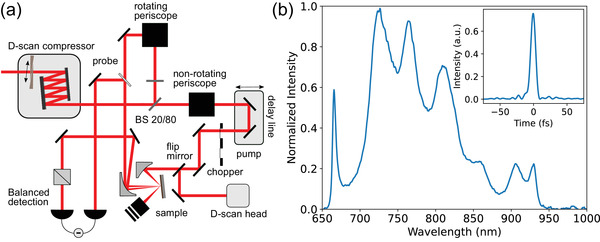
a) Schematics of the optical set‐up for degenerate pump‐probe measurements. b) Spectrum of the sub‐10 fs optical pulse used in the measurements. Inset shows the temporal shape of a compressed pulse.

The transient optical measurements were performed with a degenerate pump‐probe setup based on a femtosecond laser (Laser Quantum Venteon) (**Figure** **6**). The laser generated trains of ≈9 fs long pulses with 80 MHz repetition rate and average power around 0.5 W; the spectrum spanned from 650 to 950 nm. The laser beam was split with the 20/80 achromatic beamsplitter into the pump and probe arms both of which were later focused onto the sample with two separate off‐axis parabolic mirrors with the reflected focal length of 5.08 cm producing a spot around 30 µm in diameter. The pump beam was routed through a mechanical delay line and chopped at a frequency of 2 kHz. The beams were cross‐polarized with respect to each other in order to suppress coherent interaction at time‐zero. Using balanced detection and a lock‐in amplifier allowed measurements of transient reflected signals ΔR/R with 10^−6^–10^−7^ accuracy. Pump and probe beams were focused in orthogonal planes making them both s‐polarized in their respective planes of incidence with the probe beam being focused at near normal incidence and the pump beam being focused at 45° angle of incidence. The setup was equipped with dispersion control (Sphere Photonics D‐Scan) which consisted of a chirp‐mirror and a glass wedge compressor. It was observed that the pump‐induced change in the reflection coefficient did not completely return to zero for the 12.5 ns time interval between pump pulses as the phonon system did not completely dissipate energy, leading to the signal which did not depend on the delay between the pump and probe pulses. Therefore, all pump‐probe curves were corrected to this background to display zero change before time‐zero.

### Pump‐Probe Spectroscopy

Spectrally resolved pump‐probe measurements (presented in Supporting Information) were performed in a non‐degenerate pump‐probe setup based laser system comprised the Light Conversion Pharos laser (wavelength 1024 nm, pulsewidth around 200 fs, average power around 5 W) and Light Conversion Orpheus HP parametric amplifier which provided a tuneable output in the desired spectral range. Measurements were performed in transmission at near‐normal incidence. One of the outputs was routed through a mechanical delay line and chopped at a frequency of 2 kHz. Lock‐in amplification and balanced detection were used to increase the signal‐to‐noise ratio.

### Calculation of Hot‐Carrier Scattering

Groundstate calculations were performed for CuS in the local density approximation (LDA) with 400 eV plane wave cutoff using (12 × 12 × 4) k‐point mesh centered at the Γ point. The results were used in the single‐shot G0W0 calculation with 200 eV cutoff to generate quasiparticle energies for 40 bands close to the Fermi level. All the calculations were performed in the open‐source GPAW code.^[^ ^66^, ^67^ ^]^ Since GW implementation in GPAW by default only stores a real part of the self‐energy, the code was modified to output an imaginary part as well. The scattering time was then obtained from the imaginary part of the self‐energy.^[^ ^49^ ^]^ The results were similar to the imaginary part of the self energy calculated with the quasiparticle self‐consistent GW approximation.^[^ ^41^ ^]^ Calculations of the carrier density profile (Figure 2, inset) were also performed in the GPAW framework using the jellium model.^[^ ^51^ ^]^

## Conflict of Interest

The authors declare no conflict of interest.

## Supporting information

Supporting Information
