# Supplementary material for: Ultrafast Carrier and Lattice Dynamics in Plasmonic Nanocrystalline Copper Sulfide Films
Source: Laser Photon Rev. 2021 Jan 21;15(3):2000346. doi: 10.1002/lpor.202000346 (PMC8408971; doi:10.1002/lpor.202000346)
Supplement: Supplementary file 1 — Supporting Information [file LPOR-15-2000346-s001.pdf]

Supporting Information
Ultrafast carrier and lattice dynamics in plasmonic nanocrystalline copper sulfide films

Anton Yu. Bykov, Amaresh Shukla, Mark van Schilfgaarde, Mark A. Green, and Anatoly V. Zayats

Department of Physics and London Centre for Nanotechnology,

King's College London, London, WS2R 2LS, UK

1. POWER DEPENDENCE OF THE PUMP-PROBE RESPONSE

Figure S1(a-b) shows the sequence of the pump-probe traces for the bulk CuS crystal and the mesoscopic CuS film measured with different powers of the pump beam. Similar to the results presented in the main text (Fig. 1a), the traces in these two cases have opposite sign, due to the different Fresnel coefficients for a bulk crystal and a mesoscopic film (refer to the discussion in the main text), and about an order of magnitude difference in the amplitude is due to larger interaction volume in the bulk crystals, related to the penetration depth of the pump beam in the material. The pump-probe traces measured for the CuS film clearly reveal the presence of the high-frequency coherent optical phonon mode which is practically not visible in the covellite single crystals (its amplitude is an order of magnidute smaller as discussed in the main text). The amplitude of both groups of pump-probe traces increases linearly with the increase in the pump power (Fig. 1b). No change in shape of the decay curves with the pump power is observed that indicates the weak excitation regime.

2. SPECTRALLY-RESOLVED PUMP-PROBE MEASUREMENTS

The results obtained in the degenerate broadband pump-probe experiments reported in the main text (Figs. 1 and 2) were combined with the complementary pump-probe spectroscopy to identify the possible contribution of the resonant interband transitions to the observed transient dynamics. The measurements were performed with both tuneable pump and tuneable probe.

For the measurements with a tuneable probe, a fixed pump wavelength of 1028 nm was

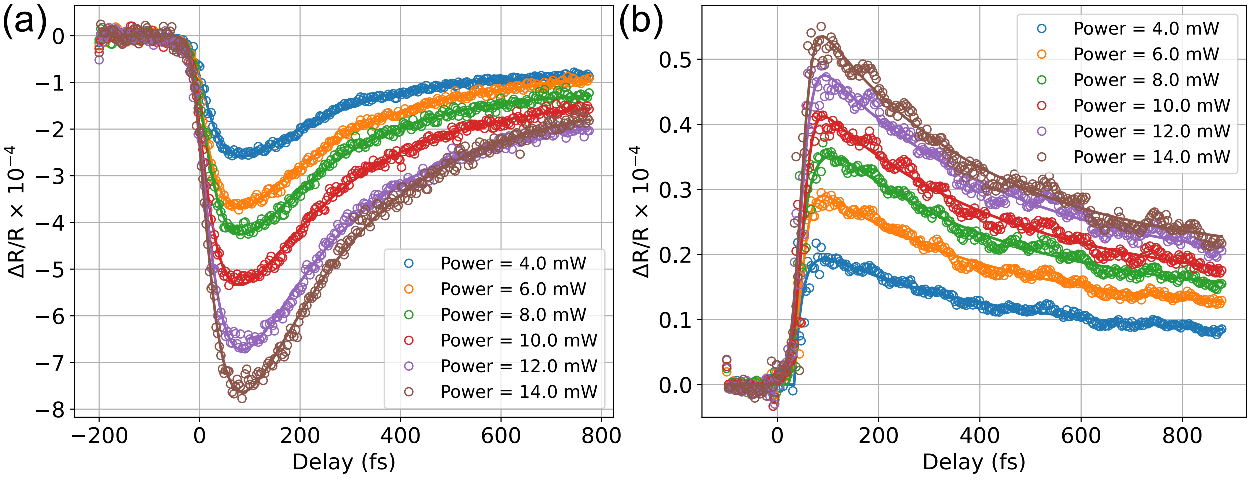

FIG. S1. Pump-probe traces for (a) a bulk CuS single crystal and (b) a mesoscopic CuS film for different pump powers.

used well below the CuS optical bandgap and a tuneable probe was in the range 700–950 nm, covering the spectral span of the few-fs broadband pulses. The amplitude of the transient response changes sign around 820 nm (Fig. S2) indicating the role of interband transitions near the Γ point of the CuS band structure (For details of band structure of CuS see [S1]). The decay times observed (broadened as compared to a previously obtained value of 200 fs due to convolution with a longer pulse cross-correlation function) do not show considerable variations with the probe wavelength (Fig. S2(b)). The observed behaviour indicates that the dynamics manifests a single decay process identified as hot-hole cooling. In order to complete the analysis, it may be noted that at the long wavelength part of the spectrum, the observed damped oscillations with the frequency around 130 GHz (Fig. S2) correspond to the acoustic vibration mode of the nanodiscs.

Similarly, in the experiments with a tuneable pump in the same wavelength range as a probe above (700–950 nm), and a fixed probe with a wavelength of 514 nm, no substantial changes in the decay times are observed either. Only a weak increase in the amplitude of the transient signal towards a long wavelength edge is observed which is consistent with the increase in the free carrier absorption in CuS upon the pump absorption. Therefore, even though the probe photons are sensitive to direct interband transitions in the spectral region of interest, hot electrons generated by these transitions do not significantly contribute to the observed dynamics. This conclusion is consistent with the high intrinsic carrier density in CuS, compared to typical semiconductors. The calculated (in-plane) dielectric constant

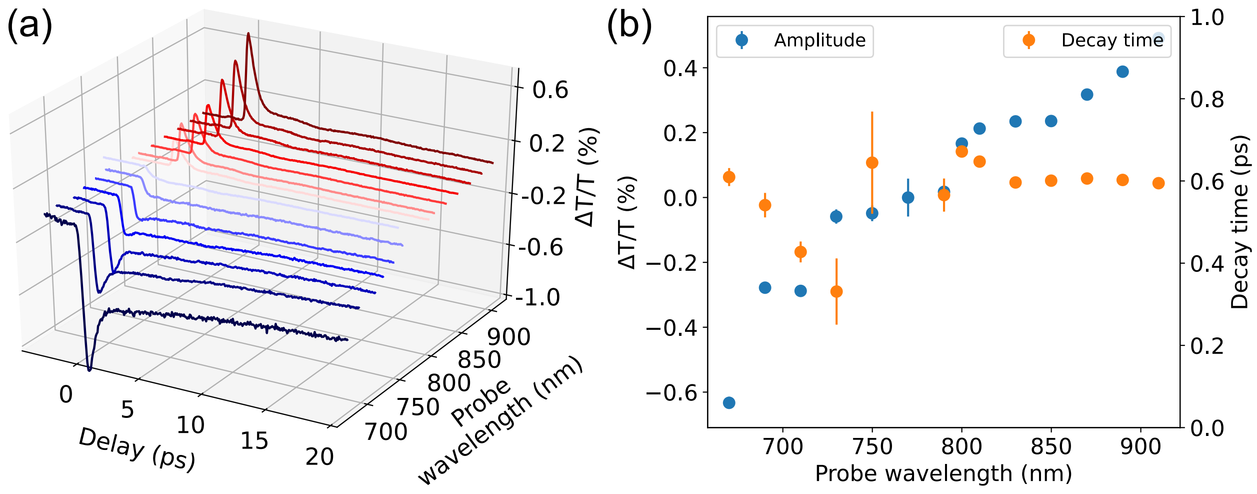

FIG. S2. (a) Spectroscopic pump-probe measurements with a 1028 nm pump and a tuneable probe beam. (b) Spectra of the amplitudes and decay times for the measurements in (a).

of CuS is in a good agreement with the Drude model for free carriers in the spectral range of interest, therefore, implying that the main absorption channel in CuS in these spectral range is free carrier absorption.

3. COHERENT OPTICAL PHONONS: SYMMETRY ANALYSIS

Covellite crystals possess a hexagonal symmetry characterized by the space group $P6_3/mmc$. The unit cell of covellite contains 14 atoms which ideally leads to 39 optical phonon modes. Since the crystal possesses the center of inversion, these modes can be explicitly divided into IR-absorption active and Raman-active. Group-theoretical classification, therefore, predicts 14 Raman active optical phonons classified as [S2, S3]: $2A_{1g}+2E_{1g}+4E_{2g}$. The full classification which includes the modes not active in the Raman scattering can be found, for example, in Ref. [S3].

If we set the crystallographic coordinate system so that x $\parallel$ $[2\bar{1}\bar{1}0]$, y $\parallel$ $[0\bar{1}10]$, z $\parallel$ [0001] $\parallel$ c-axis, then the corresponding Raman tensors for the phonon modes will take form as in Table S1.

Since the bulk crystals are always cleaved in the [0001] direction and the disk-shaped nanocrystallites are formed with the disk surface perpendicular the c-axis, the crystallographic z-axis is pointing perpendicular to the surface normal in all the studied samples.

TABLE S1. Raman tensors for Raman active optical phonon modes in covellite.

A_{1g}	E_{1g}	E_{2g}
$\begin{pmatrix} a & 0 & 0 \\ 0 & a & 0 \\ 0 & 0 & b \end{pmatrix}$	$\begin{pmatrix} 0 & 0 & 0 \\ 0 & 0 & c \\ 0 & c & 0 \end{pmatrix}$ and $\begin{pmatrix} 0 & 0 & -c \\ 0 & 0 & 0 \\ -c & 0 & 0 \end{pmatrix}$	$\begin{pmatrix} d & 0 & 0 \\ 0 & -d & 0 \\ 0 & 0 & 0 \end{pmatrix}$ and $\begin{pmatrix} 0 & -d & 0 \\ -d & 0 & 0 \\ 0 & 0 & 0 \end{pmatrix}$

Within the framework of the impulsive stimulated Raman scattering (ISRS), the driving force F_Q of phonon vibration is described by [S4]

$$F_Q = \left(\frac{\partial \alpha}{\partial Q}\right)_{ij} E_i E_j^* \,, \tag{1}$$

where the term in brackets represents the components of the Raman tensor and Q is a phonon coordinate. The validity of such simple description is usually limited to transparent media where the components of the Raman tensor are real values. In opaque materials, more sophisticated approach leads to additional terms that are displacive in nature and proportional to the time integral of the optical pulse. Nonetheless, the symmetry properties of excitation and detection of coherent phonons can still be inferred from the symmetry of the Raman tensors [S4]. Therefore, without the loss of generality, we might assume ISRS as an excitation mechanism here. Since the laboratory coordinate system (in which the fields are defined) and the crystallographic coordinate system share the z-axis and both the pump and probe beams are s-polarized, we immediately obtain from Eq. 1 that the excitation of E_{1g} modes is forbidden by symmetry. The excitation of the E_{2g} modes, however, is allowed. Using the Raman tensors from Table S1 and Eq. 1, it is easy to show that the two corresponding modes have symmetry similar to E_g modes in Bi and E_2 mode in GaN and are excited following a $\cos 2\phi (\sin 2\phi)$ dependence, where the angle ϕ is an angle between the electric field of the pump and x-axis of the crystallographic coordinate system [S5, S6]. Finally, detection of coherent phonon is described by another component of the Raman tensor and the change in reflectivity is proportional to

$$\Delta R = E_i^{(out)} \left(\frac{\partial \alpha}{\partial Q}\right)_{ij} E_j^{(in)*} \,. \tag{2}$$

Both of A_{1g} and E_{2g} modes can be observed in the change in the transient reflection but visibility of E_{2g} modes is usually enhanced when anisotropic detection schemes are implemented

[S5]. Nonetheless, we did not observe the low frequency mode in the CuS single crystals even with anizotropic detection. Finally, the symmetry of the observed phonon mode can be checked by rotating the polarization of the probe beam with respect to the polarization of the pump beam. Indeed, if we assume that the pump polarization is rotated with respect to the crystallographic coordinate system by the angle ϕ_1 and the probe by ϕ_2, we immediately obtain from Eqs. (1, 2) for E_{2g}:

$$\begin{aligned}\Delta R &= d^2|e|^2|E|^2(\cos 2\phi_1 \cos^2\phi_2 - \cos 2\phi_1 \sin^2\phi_2 + 2\sin 2\phi_1 \sin\phi_2 \cos\phi_2) = \\ &= d^2|e|^2|E|^2 \cos 2(\phi_1 - \phi_2) \propto \cos 2\Delta\phi\end{aligned} \tag{3}$$

Therefore, regardless of the orientation of the laboratory coordinate system with respect to the crystallographic one, "transversal" E_{2g} optical phonons will follow the cosine dependence as a function of a relative angle between polarizations of the pump and probe beams. It can be shown in a straightforward way that no such dependence is expected for the fully-symmetric A_{1g} modes.

[S1] R. M. Córdova-Castro, M. Casavola, M. van Schilfgaarde, A. V. Krasavin, M. A. Green, D. Richards, and A. V. Zayats, ACS Nano **13**, 6550 (2019).

[S2] M. I. Aroyo, J. M. Perez-Mato, C. Capillas, E. Kroumova, S. Ivantchev, G. Madariaga, A. Kirov, and H. Wondratschek, Zeitschrift für Kristallographie-Crystalline Materials **221**, 15 (2009).

[S3] M. Ishii, K. Shibata, and H. Nozaki, Journal of Solid State Chemistry **105**, 504 (1993).

[S4] T. E. Stevens, J. Kuhl, and R. Merlin, Phys. Rev. B **65**, 144304 (2002).

[S5] K. Ishioka, M. Kitajima, and O. V. Misochko, Journal of Applied Physics **100**, 093501 (2006).

[S6] K. J. Yee, K. G. Lee, E. Oh, D. S. Kim, and Y. S. Lim, Phys. Rev. Lett. **88**, 105501 (2002).
